# Medical-grade honey does not reduce skin colonization at central venous catheter-insertion sites of critically ill patients: a randomized controlled trial

**DOI:** 10.1186/cc11849

**Published:** 2012-10-30

**Authors:** Paulus H Kwakman, Marcella C Müller, Jan M Binnekade, Johannes P van den Akker, Corianne A de Borgie, Marcus J Schultz, Sebastian A Zaat

**Affiliations:** 1 Department of Microbiology, Center for Infection and Immunity Amsterdam (CINIMA), Academic Medical Center, University of Amsterdam, Meibergdreef 15, Amsterdam, 1105 AZ, the Netherlands; 2 Department of Intensive Care Medicine, Academic Medical Center, University of Amsterdam, Meibergdreef 15, Amsterdam, 1105 AZ, the Netherlands; 3 Laboratory of Experimental Intensive Care and Anesthesiology (L·E·I·C·A), Academic Medical Center, University of Amsterdam, Meibergdreef 15, Amsterdam, 1105 AZ, the Netherlands; 4 Department of Intensive Care, Erasmus Medical Center, 's-Gravendijkwal 230, Rotterdam, 3015 CE, the Netherlands; 5 Clinical Research Unit, Academic Medical Center, University of Amsterdam, Meibergdreef 15, Amsterdam, 1105 AZ, the Netherlands

## Abstract

**Introduction:**

Catheter-related bloodstream infections (CRBSIs) associated with short-term central venous catheters (CVCs) in intensive care unit (ICU) patients are a major clinical problem. Bacterial colonization of the skin at the CVC insertion site is an important etiologic factor for CRBSI. The aim of this study was to assess the efficacy of medical-grade honey in reducing bacterial skin colonization at insertion sites.

**Methods:**

A prospective, single-center, open-label randomized controlled trial was performed at the ICU of a university hospital in The Netherlands to assess the efficacy of medical-grade honey to reduce skin colonization of insertion sites. Medical-grade honey was applied in addition to standard CVC-site dressing and disinfection with 0.5% chlorhexidine in 70% alcohol. Skin colonization was assessed on a daily basis before CVC-site disinfection. The primary end point was colonization of insertion sites with >100 colony-forming units at the last sampling before removal of the CVC or transfer of the patient from the ICU. Secondary end points were quantitative levels of colonization of the insertion sites and colonization of insertion sites stratified for CVC location.

**Results:**

Colonization of insertion sites was not affected by the use of medical-grade honey, as 44 (34%) of 129 and 36 (34%) of 106 patients in the honey and standard care groups, respectively, had a positive skin culture (*P *= 0.98). Median levels of skin colonization at the last sampling were 1 (0 to 2.84) and 1 (0 to 2.70) log colony-forming units (CFUs)/swab for the honey and control groups, respectively (*P *= 0.94). Gender, days of CVC placement, CVC location, and CVC type were predictive for a positive skin culture. Correction for these variables did not change the effect of honey on skin-culture positivity.

**Conclusions:**

Medical-grade honey does not affect colonization of the skin at CVC insertion sites in ICU patients when applied in addition to standard disinfection with 0.5% chlorhexidine in 70% alcohol.

**Trial registration:**

Netherlands Trial Registry, NTR1652.

## Introduction

Central venous catheters (CVCs) are indispensable for the treatment of critically ill patients. Intensive care unit (ICU) patients frequently have catheter-related bloodstream infections (CRBSIs), a complication with high morbidity and mortality, and increased resource utilization [1]. Based on a conservative estimate, the total extra costs attributable to CRBSIs are almost $1 billion every year in the United States alone [2,3].

CRBSIs are caused mostly by bacteria originating from the skin at CVC-insertion sites [4,5]. Coagulase-negative staphylococci, *Staphylococcus aureus*, enterococci, and various Gram-negative bacteria are responsible for the majority of CRBSI episodes in critically ill patients [6]. Despite routine disinfection, approximately 30% of insertion sites become colonized [4,5,7]. Prophylactic use of topical antibiotics has the potential to reduce the colonization of catheter-insertion sites and thereby to reduce the incidence of CRBSIs [8], but its large-scale use is strongly discouraged because of the increased risk of resistance development [6]. Therefore, alternative antimicrobial strategies to reduce colonization of insertion sites are urgently needed.

Honey has been used as an antimicrobial preparation for thousands of years for treatment of wounds and prevention of infections. Clinical investigations in mild- to moderate-burn patients show that honey strongly reduces bacterial colonization of the skin and accelerates wound healing compared with silver sulfadiazine treatment [9,10]. As honey exerts its bactericidal activity by various mechanisms, the risk for resistance development may be considered negligible [11]. Revamil is a CE-marked, γ-irradiated medical-grade honey with potent *in vitro *bactericidal activity against a broad spectrum of antibiotic-resistant bacteria. The bactericidal activity of this honey is based on its high sugar concentration, the presence of hydrogen peroxide produced in diluted honey by the glucose oxidase enzyme, the antimicrobial peptide bee defensin-1, methylglyoxal, and the low pH [12].

Because Revamil effectively reduces skin colonization in healthy human volunteers [13], we hypothesized that application of this honey could reduce skin colonization at catheter insertion sites in critically ill patients. In a randomized controlled trial, we assessed the efficacy of daily topical application of this medical-grade honey to reduce skin-culture positivity at the insertion sites of ICU patients.

## Materials and methods

### Ethical approval and informed consent

The Medical Ethics Committee of the Academic Medical Center, Amsterdam, The Netherlands, reviewed and approved the study protocol. The trial was registered at The Netherlands Trial Registry (NTR number 1652). Informed consent was obtained from all patients or their next of kin before inclusion into the study.

### Patients

The study was performed at the ICU of the Academic Medical Center, Amsterdam, The Netherlands. Patients were eligible if they had a CVC *in situ *for less than 48 hours that was inserted in the ICU, in the operating theater, or in an emergency department with all appropriate sterile barriers. Only patients with an expected ICU stay of >48 hours were eligible for participation. Patients with skin disorders associated with an increased risk of skin colonization, patients using long-term immunosuppressive medication, pregnant patients, and moribund patients were excluded.

### Randomization

A randomization list was generated by using ALEA software with a coin-bias factor of 5 and a biased-coin threshold of 2. Allocation was concealed by using numbered envelopes. The details of the allocations were unknown to any of the investigators.

### Medical-grade honey

For the honey-treated group, the entire content of a syringe containing 2 g of Revamil (Bfactory Health Products, Rhenen, The Netherlands) was applied to the gauze CVC dressing.

### Study protocol

The study was a prospective, open-label, randomized controlled trial. After informed consent was obtained, the first newly inserted catheter was included in the study. Inclusion of only one CVC per patient was permitted in this study. Patients who were enrolled were randomly assigned to treatment with honey or standard catheter care. At inclusion, the skin surrounding the insertion site was disinfected, according to standard procedures, with 0.5% chlorhexidine in 70% alcohol. A sterile gauze dressing with or without honey was applied to the insertion site, and the gauze was covered with a transparent Tegaderm dressing (3M Health Care, St. Paul, MN, USA).

During the entire study period, an area of approximately 3 × 3 cm of the skin at the insertion site was sampled daily with a cotton swab moistened with sterile normal saline. Moistened cotton swabs are a commonly used method to determine the level of skin colonization [7,14-17]. Subsequently, the skin was disinfected according standard procedures, as described earlier, and new gauze with or without honey was applied. This procedure was continued either until the CVC was removed, until the patient was discharged from the ICU, or until the patient died, whichever came first.

### Microbiologic analysis

After sampling of the skin, the tip of the cotton swab was transferred to a 1.5-ml tube containing 0.5 ml of sterile saline. The tube was sonicated in a waterbath for 30 seconds and subsequently vortexed for 10 seconds. The cotton tip was removed, and the sonicate was quantitatively cultured on blood agar plates at 37°C for 2 days.

### Primary and secondary end points

The primary outcome was colonization of skin surrounding the insertion site with >100 CFU/swab at the last sampling. Such a level of skin colonization during catheter use is a major risk factor for CRBSI [15,17,18]. Secondary outcomes were the level of colonization at the last sampling and stratification of these outcomes by catheter location, duration of catheter stay, ICU length of stay, and ICU and hospital mortality.

### Microbial identification

Microorganisms cultured from the skin of patients with a positive primary end point were identified by using VITEK-2 (BioMérieux, Boxtel, the Netherlands) and classic biochemical determination. We also collected clinical microbiologic data of culture-positive blood samples from patients taken during their inclusion in the study. Blood samples were drawn only for clinical diagnosis and not for study purposes.

### Power calculation

We aimed for a clinically relevant reduction in skin-colonization frequency of 50%, which, based on available literature, meant a reduction from 30% to 15% culture positivity [4,5,7]. A two-group χ^2 ^test with a 0.050 two-sided significance level and an 80% power to detect the difference between a Group 1 proportion of 0.3 and a Group 2 proportion of 0.15 required a sample size of 120 patients for each group.

### Statistical analysis

Continuous normally distributed variables were expressed by their mean and standard deviation or, when not normally distributed, as medians and their interquartile ranges. Categoric variables were expressed as *n *(%). To test groups, Student *t *tests were used; if continuous data were not normally distributed, the Mann-Whitney *U *test was used. Categoric variables were compared with the χ^2 ^or Fisher Exact tests.

In addition, we aimed to quantify the net effect of the application of medical-grade honey on the colonization of insertion sites, controlling for other variables. Exploration of interaction (effect modification) and confounding was considered methodologically relevant for this approach. Therefore, we first focused on the crude (uncorrected) effect of honey (independent variable) on colonization (dependent variable). Then statistical and clinically relevant covariates were added as an interaction term. If the interaction term appeared to be significant (*P *< 0.05), this would indicate that the relation between the honey and colonization could be different for various levels of the covariate. This indicates the need for separate models for the levels of the covariate.

As a significant interaction was not found, the model was examined for confounding. Confounding was defined as ≥10% change in the coefficient of the central determinant (honey) as a consequence of adding a covariate.

To analyze for an effect of honey or other variables on the level of skin colonization over time, a mixed-model repeated measures method was used to deal with unbalanced data due to different numbers of cultures per patient and missing values. We used a random intercept and random slopes model. Treatment group, catheter type, and catheter location were fixed covariates.

Statistical significance was considered to be at *P = *0.05. When appropriate, statistical uncertainty was expressed by the 95% confidence levels. Analysis was performed with SPSS version 18.2.

## Results

### Patients

**Figure 1:**
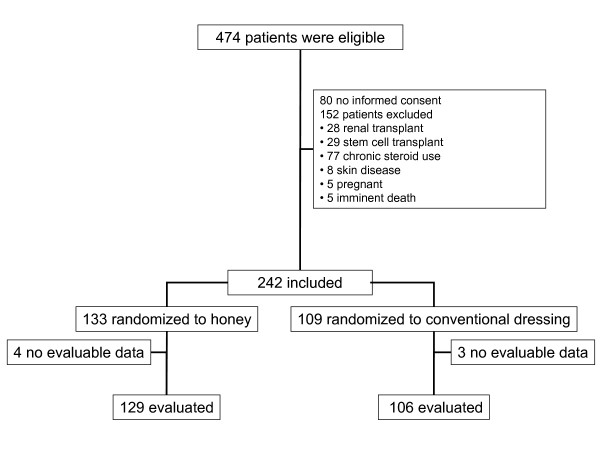
**CONSORT flow diagram**.

In total, 242 patients were enrolled, of whom 133 and 109 were assigned to the honey and control group, respectively (Figure 1). For seven participants (four in the honey group and three in the control group), no data were obtained, because these patients were lost to follow-up before a skin swab was taken. Baseline characteristics of evaluated patients are described in Table 1.

**Table 1:** Baseline patient and catheter characteristics

	Honey	Control
Patients, *n *(%)	129 (54.9)	106 (45.1)
Male, *n *(%)	68 (53)	69 (65)
Age, mean (SD)	63 (14)	63 (15)
APACHE II score, mean (SD)	21 (7)	22 (7)

Admission type, *n *(%)		

Medical	71 (56)	58 (55)
Surgical urgent	30 (23)	29 (27)
Surgical elective	27 (21)	19 (18)
Confirmed infection at admission, *n *(%)	32 (25)	26 (25)
Immunosuppressive medication, *n *(%)	7 (5)	6 (6)
Neutropenic, *n *(%)	8 (6)	3 (3)

Catheter location, *n *(%)		

Femoralis	60 (46)	65 (61)
Jugularis	45 (43)	29 (27)
Subclavia	14 (11)	12 (11)

Catheter type		

CVVH	32 (25)	26 (25)
Swan-Ganz	7 (5)	3 (3)
One lumen	0	2 (2)
Three lumens	88 (68)	72 (68)
Four lumens	1 (1)	0
CVVH with an extra lumen	1 (1)	2 (2)
Side port	0	1 (1)

### The effect of honey on skin colonization

All included catheters were sampled for the consecutive days that the patients resided in the ICU. Culture results for all samples of the honey and control group are presented in Additional file 1. In both the honey-treated group and the control group, 34% of the patients had a positive skin culture (defined as >100 CFUs) at the last sampling point (Table 2). Median (IQR) levels of skin colonization at the last sampling were 1 (0 to 2.84) and 1 (0 to 2.70) log CFU/swab for the honey and control groups, respectively (*P *= 0.94). In the honey group, more jugular catheters were included compared with the control group, whereas in the latter group, more femoral catheters were included (Table 1). However, stratification according to CVC location did not reveal significant differences in the percentage of positive skin cultures or in quantitative levels of colonization between the honey and control groups (Table 2).

**Table 2:** Skin-colonization outcomes

	Honey *n *= 129	Control *n *= 106	*P *value
Last culture positive, *n *(%)	44 (34)	36 (34)	0.98
Log CFU/swab, median (IQR)	1 (0-2.84)	1 (0-2.70)	0.94

Last culture, stratified for catheter location			

Femoralis, *n *(%)	22 (37)	25 (38)	0.21
Jugularis, *n *(%)	20 (44)	10 (34)	0.17
Subclavia, *n *(%)	2 (14)	1 (8)	0.68

Log CFU/swab, stratified for catheter location			

Femoralis, median (IQR)	1.3 (0-2.95) *n *= 60	1.30 (0-2.73) *n *= 65	1.0
Jugularis, median (IQR)	0 (0-3.2) *n *= 55	1 (0-2.7) *n *= 29	0.89
Subclavia, median (IQR)	0 (0-0) *n *= 14	0 (0-0) *n *= 12	0.90

Last culture is defined as the last sampling time point before catheter removal, transfer of patient from the ICU, or death.

Median ICU length of stay was 9 (4 to 18) and 9 (5 to 16) days for patients in the honey-treated and control groups, respectively (*P *= 0.67). Median duration of catheter use was 5 (3 to 7) days for both study groups (*P *= 0.32). ICU mortality was 24% and 23% in the honey and control groups, respectively (*P *= 0.88), and total hospital mortality was 36% and 34% for the respective groups (*P *= 0.89). Reasons for removal of the CVC were also not significantly different for both study groups (Table 3).

**Table 3:** Reasons for catheter removal

	Honey *n *= 129	Control *n *= 106	*P *value
No longer needed, *n *(%)	54 (42)	39 (37)	0.42
Suspected infection, *n *(%)	20 (16)	15 (14)	0.77
Dysfunction, *n *(%)	5 (4)	4 (4)	0.97
Patient died, *n *(%)	19 (15)	18 (17)	0.64
Patient discharged, *n *(%)	31 (24)	30 (28)	0.46

Multivariate logistic regression was performed to obtain insight into the role of baseline characteristics as predictors of positive skin cultures. Duration of CVC use, CVC location, CVC type, and gender appeared to be statistically associated (*P *≤ 0.10) with positive skin cultures. We found no interaction effect of any of the covariates in multivariate analysis. Although the statistically associated covariates did qualify as confounders (that is, adding these variables to the logistic model changed the beta factor of the crude relation between study group and positive skin cultures by more than 10%), the primary predictor (medical-grade honey) remained nonsignificant (that is, OR, 1.01 (0.59 to 1.73); *P *= 0.98.

A mixed-model repeated measures method was used to assess whether a difference existed in the level of skin colonization over time between the honey and control groups. Honey did not have a significant effect on quantitative level of skin colonization (*P *= 0.37). The duration of catheter use significantly affected skin colonization; the positive value of the regression coefficient showed that the log CFU/swab count increased for successive samplings. Furthermore, a significant increase was found in log CFU/swab for single-lumen catheters over time (*P *= 0.03). Catheters inserted at the subclavian site showed a significant decrease in log CFU/swab over time (*P *= 0.01).

### Microorganisms cultured from skin and blood

Coagulase-negative staphylococci (CoNSs) were the most frequently isolated bacteria at insertion sites, accounting for 62% and 60% of all isolated microorganisms in the honey and control groups, respectively. Enterococci were isolated in 30% of positive skin cultures for both study groups. Streptococci, micrococci, *Klebsiella *spp., and *Pseudomonas *spp. were responsible for the remaining positive skin swabs. Eleven (16%) of 70 and six (13%) of 48 (*P *= 0.79) blood samples from patients in the honey-treated and control groups, respectively, were positive for microbial growth. In the honey-treated group, seven blood cultures were positive for CoNS, two for enterococci, one for both CoNS and enterococci, and one for *Staphylococcus aureus*. In the control group, three blood cultures were positive for CoNS, one for both CoNS and enterococci, one for *Pseudomonas aeruginosa*, and one for *Candida glabrata *(control group).

## Discussion

Daily application of Revamil medical-grade honey did not reduce the frequency of positive skin cultures of CVC insertion sites of critically ill patients. Gender, duration of CVC use, CVC location, and CVC type were predictive for a positive culture, which is in agreement with several publications on independent risk factors for catheter-site colonization and catheter-related infections [15,18,19]. After correction for these variables, no effect of honey on frequency of positive skin cultures was detected in a multivariate logistic regression model. No differences were observed in the secondary end points, which were quantitative level of colonization of CVC sites, microbiologic outcomes for different catheter locations, ICU length of stay, duration of catheter use, and ICU and hospital mortality.

Large-scale prophylactic application of antibiotics to prevent CRBSIs is strongly discouraged because of the risk for development of antibiotic resistance [6]. Because no resistance against honey has ever been reported, honey could be a very interesting alternative to antibiotics for topical applications. To the best of our knowledge, the current study is the first in which honey as an antimicrobial agent was applied to ICU patients.

Honey was previously applied to exit sites of tunneled, cuffed, internal jugular CVCs by Johnson *et al. *[11]. These investigators showed that mupirocin significantly reduced the frequency of catheter-related bacteremia and increased time to first bacteremia for patients undergoing hemodialysis carrying such catheters [20]. In a follow-up study, they showed that insertion-site care with a manuka honey (Medihoney; Medihoney Pty Ltd, Brisbane, Australia) was as effective as mupirocin in terms of rates of catheter-related bacteremia and infection-free survival time [11]. Although the latter study was not adequately powered to demonstrate equivalence between honey and mupirocin, the results indicate that thrice-weekly application of Medihoney to tunneled, cuffed hemodialysis catheters reduces the risk of catheter-related infections. This strongly suggests that the applied honey reduced colonization of the catheter-insertion sites.

Several differences between the present trial and the study by Johnson *et al*. may explain the different effects of honey. Whereas Johnson *et al*. studied the application of Medihoney to the insertion site of surgically placed, tunneled, cuffed catheters for long-term use in patients with renal failure, we applied Revamil honey to the insertion site of nontunneled, short-term CVC in critically ill patients. The modes of application of honey by Johnson *et al*. and in our study were essentially the same. They directly applied honey to the skin and covered it with a Primapore dressing consisting of an absorbent pad and a fixative layer. We applied honey on a gauze dressing, placed the gauze on the catheter-insertion site with the honey facing the skin, and covered the gauze with a transparent plaster to keep it in place. Whereas Johnson *et al*. studied hemodialysis outpatients, we studied critically ill patients. Critical illness increases capillary permeability, often accompanied by leakage of edema fluid along CVC insertion sites [21]. Indeed, we frequently observed absorption of large amounts of exudate by the gauze. Due to this leakage of fluid and the use of nontunneled catheters, the honey applied in our study might have been more strongly diluted than that in the study by Johnson *et al. *[11].

Differences between our trial results and those of Johnson *et al*. may also lie in the compositions of the honeys used. Honeys differ in their composition of antimicrobial compounds, Revamil relying particularly on hydrogen peroxide production and the antimicrobial peptide bee defensin-1, and manuka honey, mostly on methylglyoxal [22]. The activity of Revamil honey against staphylococci and enterococci particularly depends on hydrogen peroxide [12]. Hydrogen peroxide is formed on dilution of honey. When Revamil honey is diluted by wound exudate on application at catheter-insertion sites, the hydrogen peroxide produced may become degraded by catalase present in this exudate. This could aggravate the loss of antibacterial activity of Revamil honey on dilution. Because manuka-based honeys like Medihoney, as used by Johnson *et al*., retain activity up to higher dilutions than Revamil honey, owing to high levels of methylglyoxal and other nonperoxide antimicrobial compounds, they are less prone to lose activity because of dilution by wound exudate [22]. The combination of possible lower levels of exudation in the study by Johnson *et al*. and the lower loss of activity of Medihoney on dilution, may thus have favored the outcome of their study. Therefore, honeys that retain antibacterial activity up to high dilutions in wound fluid might be more effective than honeys like Revamil for applications at sites of high exudation.

In this study, we used moistened cotton swabs to assess skin colonization. This is one of the most commonly used methods to assess skin colonization [14,15,17,18]. We considered this method more appropriate than the contact-plate method [23], because the contact-plate method requires the sampled skin surface to be flat. This was not the case in our study because of the presence of the catheter and the anatomy of the jugular and femoral catheter locations. Moreover, the very high level of catheter-site colonization, as observed for a substantial number of patients in our study, would have caused confluent growth when using the contact plate method and would thus have impeded quantification of the colonizing microorganisms. Thus, skin sampling using moistened cotton swabs was the method of choice in our study.

A level of colonization of approximately 100 CFU per 10 cm^2 ^of skin cultured by using a moistened cotton swab is frequently used to define positive skin cultures [14,15,17]. This level of colonization is actually reported as a major risk factor for catheter-related bloodstream infection [18]. We therefore decided to define a level of 100 CFU as a clinically relevant criterion for a positive skin culture.

We chose to perform daily dressing changes to allow frequent replacement with fresh honey to maximize the possibility to achieve an effective antibacterial treatment. Daily dressing change, however, is not routinely performed at our ICU. Guidelines regarding frequency of dressing change and type of dressing to be used are still under debate [18].

Maki and Ringer [24] showed that replacement of gauze dressings every other day results in slightly lower levels of colonization of catheter sites compared with a situation in which the dressing remains in place for the lifetime of the catheter; the differences in levels of skin colonization between these dressing regimens were, however, only minute. The authors concluded that it would not be cost effective to redress catheters at periodic intervals. More recently, also for application of transparent polyurethane dressings and for chlorhexidine-impregnated sponges, it was shown that less-frequent catheter-dressing changes do not increase the risk for catheter infection, while significantly reducing patient discomfort and costs [25,26]. On the basis of the studies described, the CDC guidelines for prevention of catheter-infection advise changing dressings at least every 7 days, but do not advise against more-frequent dressing change [27]. The results of our trial, however, should be interpreted in the context of daily dressing changes.

Several weaknesses exist in the design of our trial. Our trial has limitations inherent to a single-center design. Moreover, this trial was performed at an ICU by using selective decontamination of the digestive tract (SDD) [28,29]. The SDD regimen apparently did not substantially affect microbial colonization of the skin at catheter-insertion sites, because we identified mainly coagulase-negative staphylococci and streptococci, bacterial species typically found on skin. Therefore, this suggests that the SDD regimen does not necessarily hamper extrapolation of our results to other centers not using SDD.

Second, we used an open-label design. It was inevitable to use an open-label design for this trial because no proper placebo for honey is available. The absence of blinding could have potentially introduced observer biases. The risk for such bias was minimized by using a clearly defined, objective outcome measure.

A substantial imbalance in numbers of patients was randomized in the two study arms, 133 in the honey group, and 109, in the control group. Such an imbalance is a potential source of bias, especially covariate imbalance. However, the highly comparable distribution of potential confounders between groups (Table 1), as well as the outcome of the multivariate logistic regression model, sufficiently showed the absence of such bias. Particularly the regression model demonstrated that neither effect modification nor confounding altered the conclusion.

It was not feasible to use CRBSI as an end point, because of its relatively low incidence. The reported incidence of CRBSI in international studies varies from 1.4 to 7.7 episodes per 1,000 catheter days [6,30,31]. Even when based on the highest incidence within this range, a sample size of about 14,000 catheter days would be required to identify a 50% reduction in incidence of CRBSI. Therefore, we used catheter-site colonization as a proxy for CRBSI, because colonization of the catheter site, with >100 CFU/swab at catheter removal, is a major risk factor for CRBSI [15,17,18]. Thirty percent of the skin swabs of catheter-insertion sites of ICU patients are reported culture positive [4,5,7]. The frequency of culture-positive skin swabs in the control group of our study was even somewhat higher (34%). Thus, our study was sufficiently powered to detect a 50% difference in the frequency of positive skin cultures between the study and control groups.

## Conclusions

Honey did not reduce microbial skin colonization at the insertion site of CVCs in ICU patients.

## Key messages

• Revamil medical-grade honey does not reduce microbial colonization around the insertion site of nontunneled, short-term CVCs of ICU patients.

• The natural variation in presence of different antibacterial compounds in honey might explain the observed differences in clinical efficacy of honey between our trial and a previous study by Johnson *et al. *[11].

• Extensive characterization of the composition of the bactericidal compounds is essential for application of medical-grade honey as a topical antimicrobial agent in modern medicine.

## Abbreviations

CFU: colony-forming unit; CoNS: coagulase-negative staphylococci; CRBSI: catheter-related bloodstream infection; CVC: central venous catheter; CVVH: continuous veno-venous hemofiltration; ICU: intensive care unit; SDD: selective decontamination of the digestive tract.

## Competing interests

The authors declare that they have no competing interests.

## Authors' contributions

PK designed and performed the study, data analysis, statistical analysis, and drafting of the manuscript; MM worked on the selection and inclusion of patients, execution of the RCT, data analysis, and drafting of the manuscript; JB, on the study design, statistical analysis, and drafting of the manuscript; JA, on the selection and inclusion of patients, and execution of the study; CB, on the data analysis and statistical analysis; MS, on conceiving of the study, study design, data analysis, and drafting of the manuscript; and SZ, on conceiving of the study, study design, data analysis, and drafting of the manuscript. All authors read and approved the manuscript for publication.

## Supplementary Material

Additional file 1 Culture results for the honey and control group over time. Catheter sites were sampled on a daily basis after inclusion of patients in the study. The numbers of positive skin cultures (shaded) and negative cultures (white) are indicated for the honey group (left bars) and for the control group (right bars) for consecutive days **(A)**, and the levels of skin colonization for consecutive days are indicated for the honey group (open circles) and for the control group (solid circles) in log CFU/swab **(B)**. Click here for file
