# Supplementary figures and images for: Medical-grade honey does not reduce skin colonization at central venous catheter-insertion sites of critically ill patients: a randomized controlled trial
Source: Crit Care. 2012 Oct 30;16(5):R214. doi: 10.1186/cc11849 (PMC3682318; doi:10.1186/cc11849)

**A**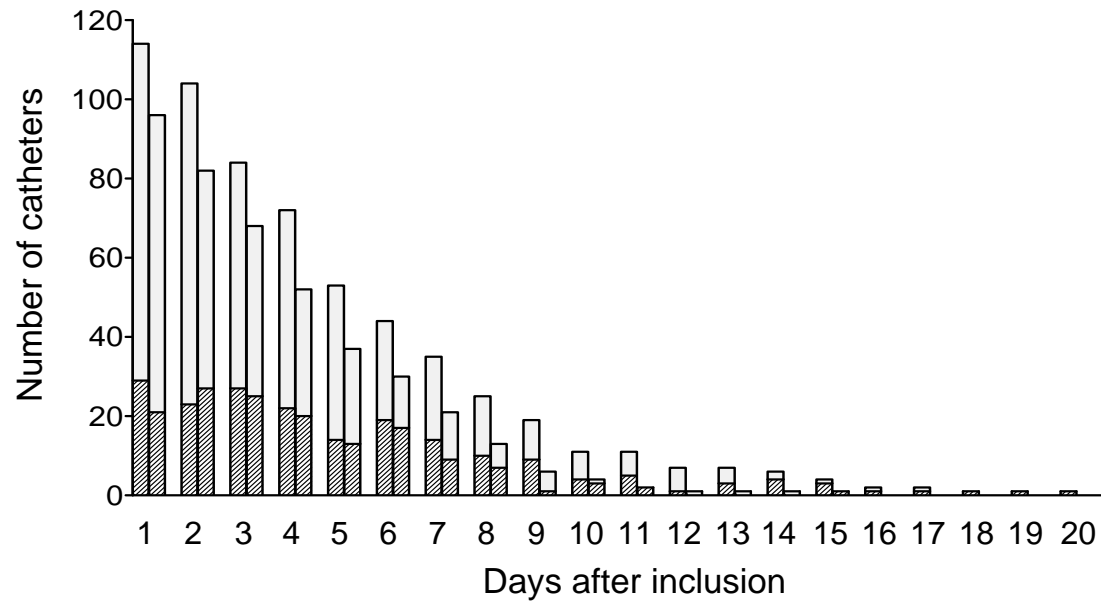**B**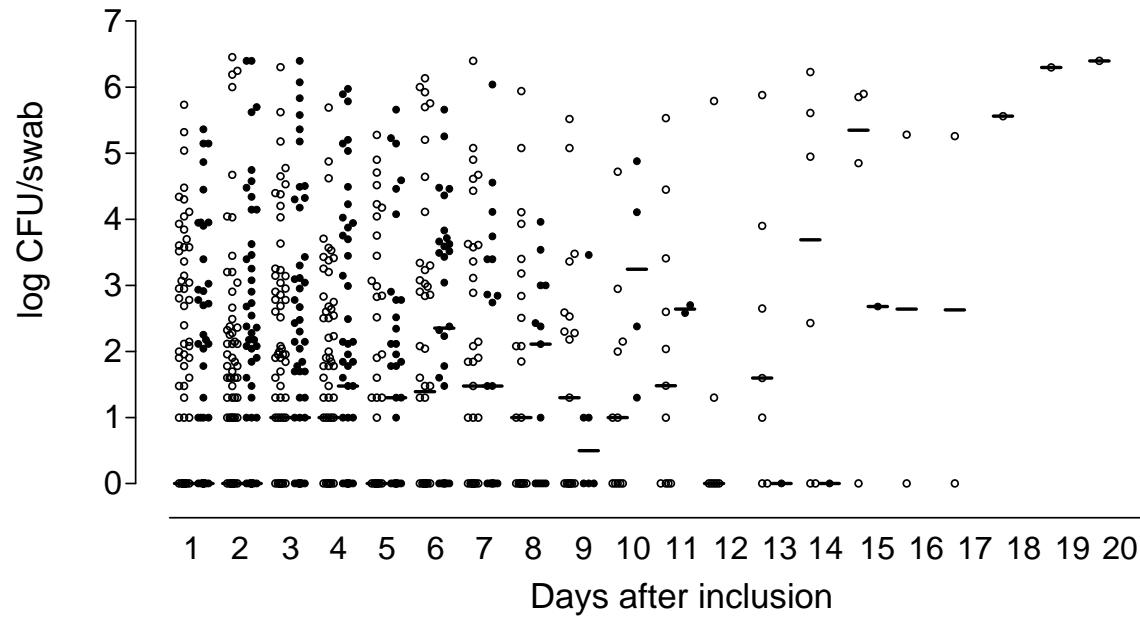

Supplement: Additional file 1 — Culture results for the honey and control group over time. Catheter sites were sampled on a daily basis after inclusion of patients in the study. The numbers of positive skin cultures (shaded) and negative cultures (white) are indicated for the honey group (left bars) and for the control group (right bars) for consecutive days (A), and the levels of skin colonization for consecutive days are indicated for the honey group (open circles) and for the control group (solid circles) in log CFU/swab (B). [file cc11849-S1.PDF]
